# Anti N-Methyl-D-Aspartate receptor antibody associated Acute Demyelinating Encephalomyelitis in a patient with COVID-19: a case report

**DOI:** 10.1186/s13256-023-03979-x

**Published:** 2023-06-16

**Authors:** Kuven Naidu, Rory Tayler

**Affiliations:** Life The Glynnwood Hospital, 33 Harrison Street, Benoni, Gauteng South Africa

**Keywords:** Anti NMDA receptor antibodies, ADEM, COVID-19, Case report

## Abstract

**Background:**

Anti N-Methyl-D-Aspartate (NMDA) receptor antibody associated ADEM is a diagnosis that was first described relatively recently in 2007 by Dalmau *et al.* The recent COVID-19 pandemic has resulted in multiple neurological complications being reported. However, there is limited data on Anti-NMDA receptor antibody associated ADEM in COVID-19 patients. Furthermore, the MRI findings in these patients have not been fully elucidated. This case report adds to the growing body of knowledge of neurological complications in COVID-19 patients.

**Case presentation:**

A 50 year old Caucasian female with no previous medical co-morbidities presented with symptoms of COVID-19 and subsequently developed neurological symptoms which included confusion, limb weakness and seizures. The patient developed marked behavioural abnormalities which required attention. She was found to have anti NMDA receptor antibodies present in a significant titres, an elevated total protein on lumbar puncture and cytotoxic magnetic resonance imaging (MRI) changes in the brain and spinal cord and was subsequently diagnosed with an anti-NMDA Receptor Antibody associated ADEM. The bilateral symmetric involvement of the corticospinal tract on MRI was considered unusual in our case. She was treated with a combination of corticosteroids and plasmapheresis which halted disease progression. Thereafter she was commenced on intravenous immunoglobulin as maintenance therapy and she has shown continuous improvement with ongoing physiotherapy.

**Conclusion:**

The recognition of COVID 19 neurological complications may be difficult in the initial disease as early symptoms of lethargy, weakness and confusion may be very nondescript. However, it is imperative that these complications are sought for as they are imminently treatable. Early institution of therapy is imperative in decreasing long term neurological consequences.

## Background

Neurological complications of COVID-19 have been steadily reported since the pandemic began in late 2019. A UK-wide surveillance study performed in the 1st half of 2020 [1] revealed a multitude of neurological complications in a 153 cases ranging from cerebrovascular events to altered mental status. Those with altered mental status were classified as having, encephalitis, unspecified encephalopathy or a psychiatric condition.

In fact, more than 80% of patients admitted with COVID-19 to a hospital in Chicago have displayed some form of neurological manifestation [2].

A case series comprising of three patients in a hospital in Minnesota, USA commented on all three patients having MRI changes and a cerebrospinal fluid (CSF) showing elevated protein indicating ADEM post COVID [3].

However, there is very limited data available on the development of an Anti- NMDA Receptor antibody associated ADEM due to COVID-19 and the treatment thereof.

## Case presentation

Ms. M, a 50 year old Caucasian female with no previous medical or psychiatric conditions, presented with a fever, cough and myalgia while on holiday by the coast in the middle of December 2020. She was admitted to hospital on the 28 December 2020 after testing positive for COVID-19. Ms. M had a family history of macular degeneration and was a non-smoker and did not use any alcohol. She developed severe nausea, and vomiting and progressive weakness of all limbs along with significant confusion and disorganised behaviour. She was not hypoxic at the time of presentation.

Her blood results ate the time revealed an elevated C-reactive protein of 50 mg/L and marginally increased D-Dimer of 0.72 ug/mL.

An MRI brain was undertaken which showed significant abnormalities consistent with a cytotoxic encephalopathy.

A lumbar puncture was performed on the 02 January 2021 and an elevated total protein (770 mg/L) was noted with no polymorphs and only 1 Red blood cell per uL. The glucose was 3.1 mmol/L (within range). The CSF IgG was 77.0 (Normal range < 34.1) and the viral screen, oligoclonal bands, TB PCR and TPHA were negative.

She was treated with a dose of pulse solumedrol 500 mg daily for 3 days and commenced on Sodium Valproate controlled release at 200 mg twice daily, Enoxaparin 40 mg daily SC and Esomeprazole 40 mg daily.

Ms. M was discharged into the care of her family after completion of the pulse therapy and returned to her home in Johannesburg. However, she continued to exhibit marked behavioural dysfunction and was also dependant on her family to perform simple activities of daily living.

She was transferred to a rehabilitation facility on the 09 January 2021 in Johannesburg where the treating practitioner noted the marked behavioural dysfunction, emotional lability, quadriparesis, left sided sensory deficits most marked in her face, tongue and extremities, slurred speech, urinary retention and cognitive impairment.

Ms. M was referred to a Johannesburg hospital on the 14 January 2021 for further neurological assessment where, in addition to above symptoms, significant cerebellar dysfunction was noted. She had evidence of truncal ataxia and brisk bilateral deep tendon reflexes with bilateral Babinski signs. There was no evidence of any meningism.

An MRI of the brain was repeated (14 January 2021) which showed improvement of the marked cytotoxic encephalopathy. There was increased signal in the region of the dentate nucleus, inferior cerebellar peduncle, corticospinal tracts at the level of the basal ganglia and midbrain with diffuse restriction evident in these areas. This was consistent with an Acute Demyelinating Encephalomyelitis (ADEM).

Anti N-methyl-d-aspartate (NMDA) receptor antibodies were performed with markedly raised titre of 1:1280 noted.

The anti neural antibodies, antiphospholipid antibodies and the antibodies against aquaporin receptors were all negative.

A diagnosis of an autoimmune anti NMDA receptor antibody encephalitis precipitated by COVID-19 was subsequently made. However further investigations were undertaken to exclude a neoplastic process, in particular, excluding ovarian tumours. A gastroscopy and colonoscopy, CT chest and abdomen, mammogram were all normal.

She was administered intravenous pulse steroid therapy, however, the clinical response was noted to be limited and she was started on intravenous immunoglobulin therapy (IVIG) 40 g daily for 5 days.

Ms. M showed subsequent clinical improvement especially with regards to her higher functions. Her strength was noted to have normalised and her cerebellar dysfunction showed much improvement but requiring of ongoing physical rehabilitation.

She was discharged back to the rehabilitation on the 26 January 2021 on Enoxaparin (Thrombosis prophylaxis) and pantoprazole where she spent the next 2 months undergoing physical therapy. She was discharged on the 19 March 2021 on Escitalopram 20 mg daily, Trazodone 50 mg daily, Esomeprazole 20 mg daily and potassium supplementation.

**Fig. 1:**
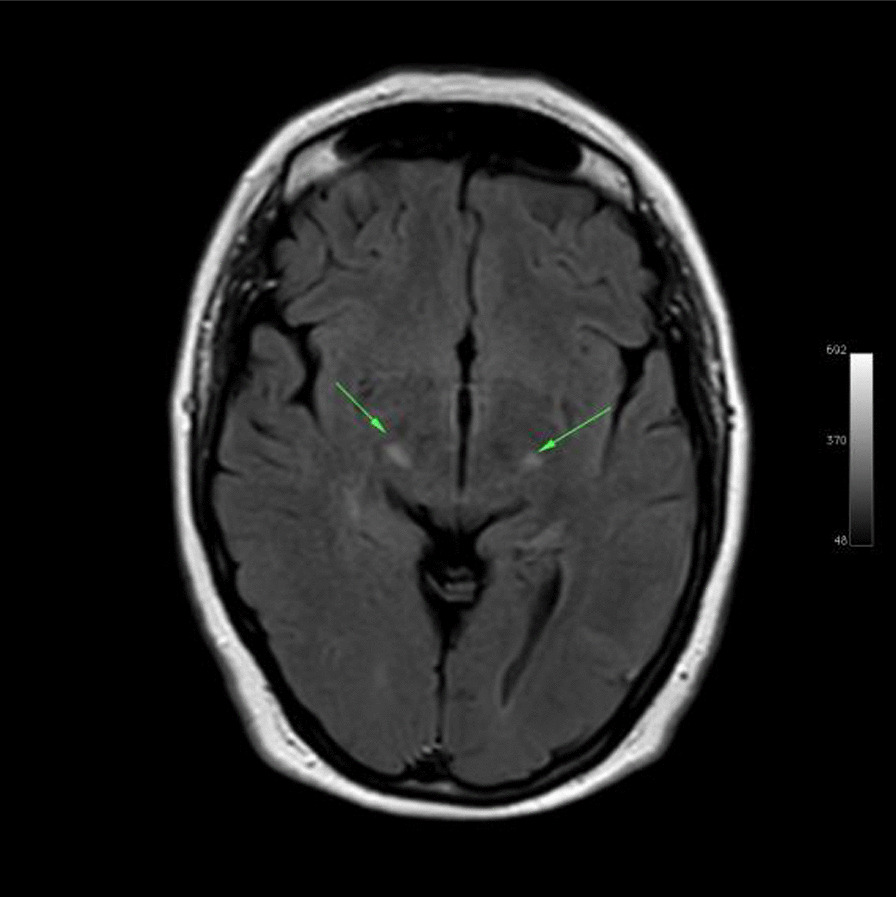
Axial lair demonstrates bilateral symmetric abnormal high signal in the corticospinal tracts. The arrows are pointing to the bilateral symmetric abnormal high signals in the corticospinal tracts

**Fig. 2:**
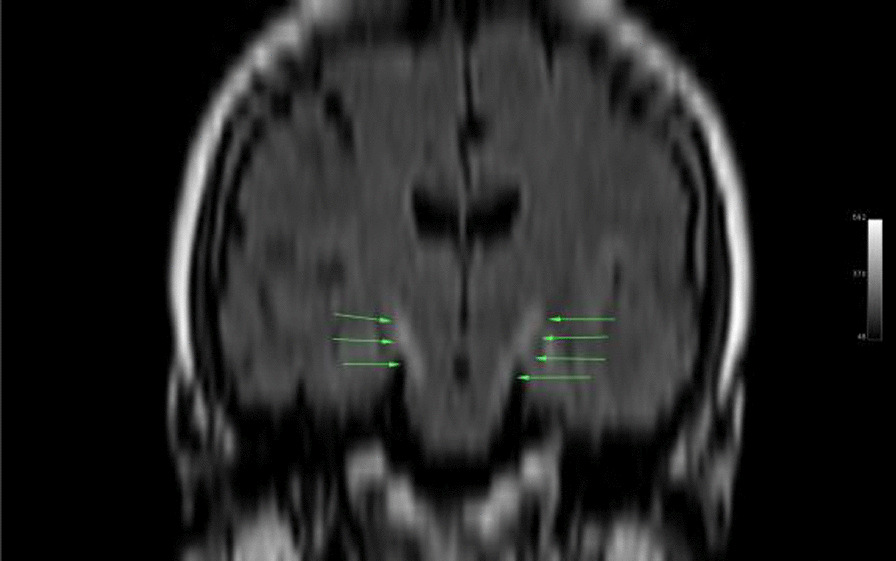
Coronal reformatted flair demonstrates bilateral symmetric abnormal high signal in the corticospinal tracts as indicated by the arrows

**Fig. 3:**
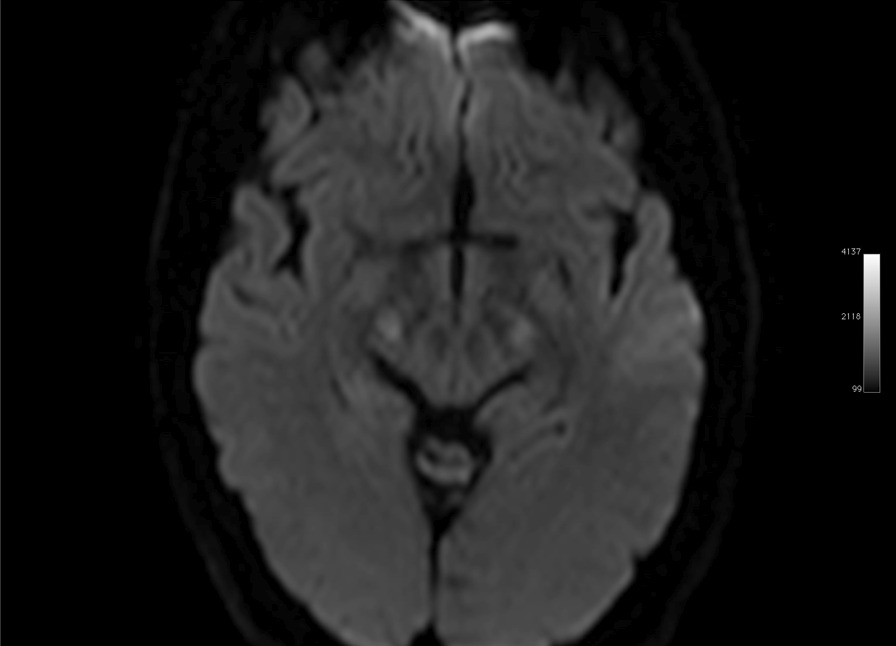
Axial DWI demonstrates T2 shine-through characteristics with high signal in the corticospinal tracts noted on the DWI with isotense signal on the apparent diffusion co-efficient map

**Fig. 4:**
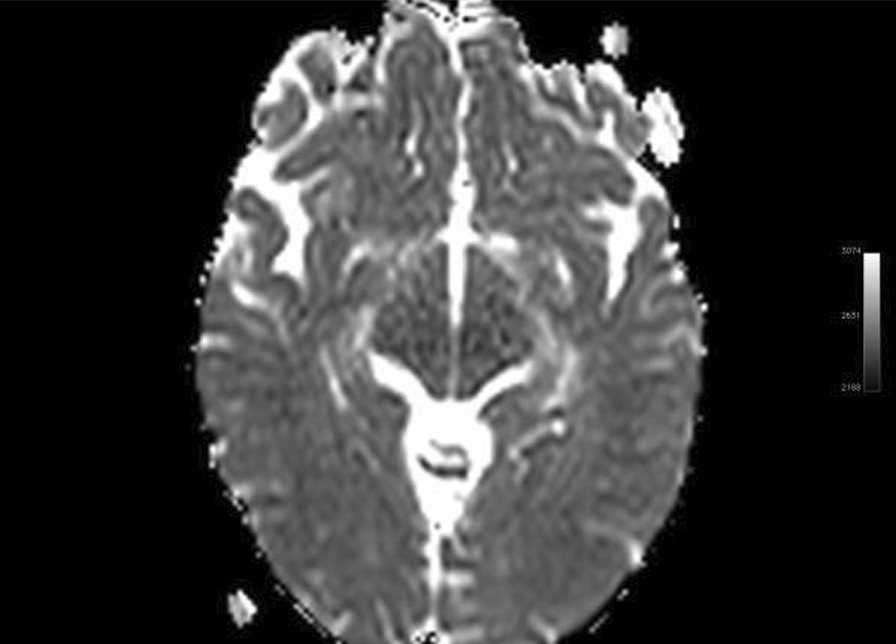
Axial ADC demonstrates T2 shine-through characteristics with high signal in the corticospinal tracts noted on the DWI with isotense signal on the apparent diffusion co-efficient map

**Fig. 5:**
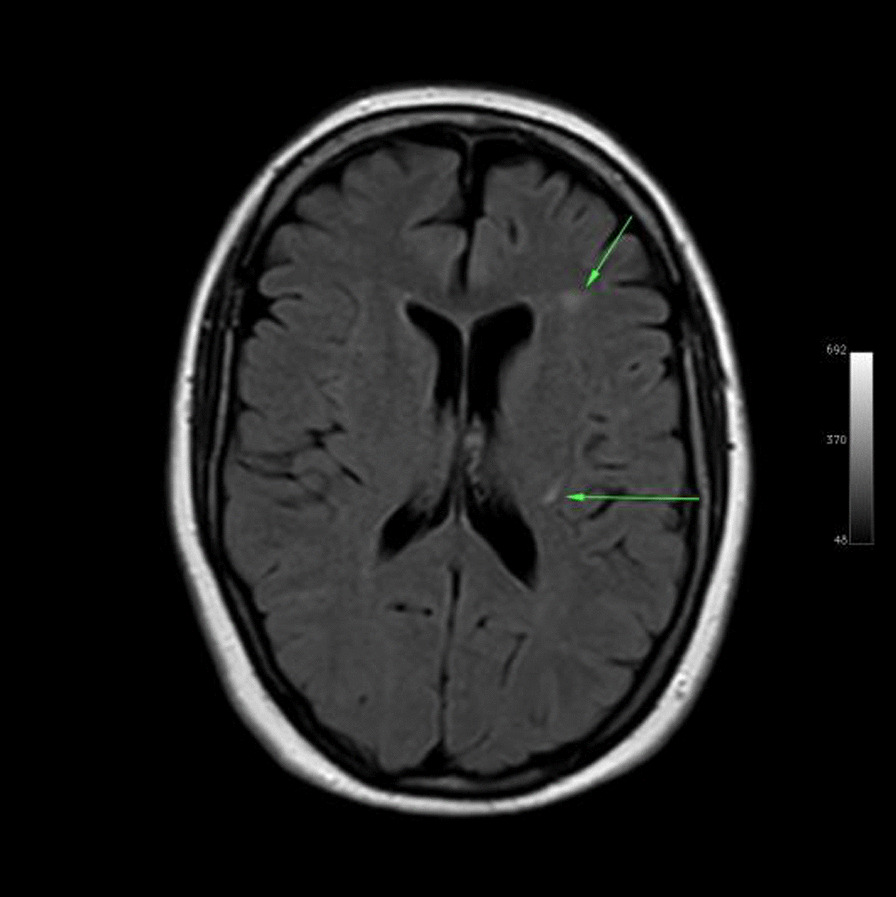
The arrows indicate the randomly distributed white matter lesions in the brain

**Fig. 6:**
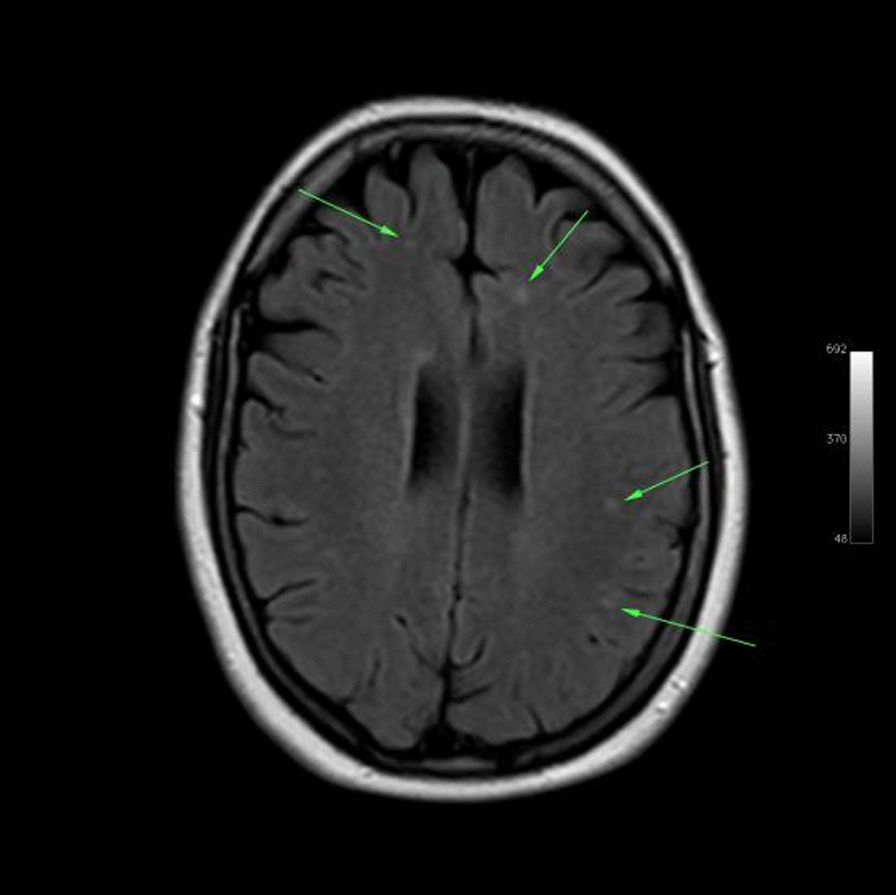
Axial flair images taken at different levels demonstrates randomly distributed white matter lesions in the rest of the brain as indicated by the arrows

**Fig. 7:**
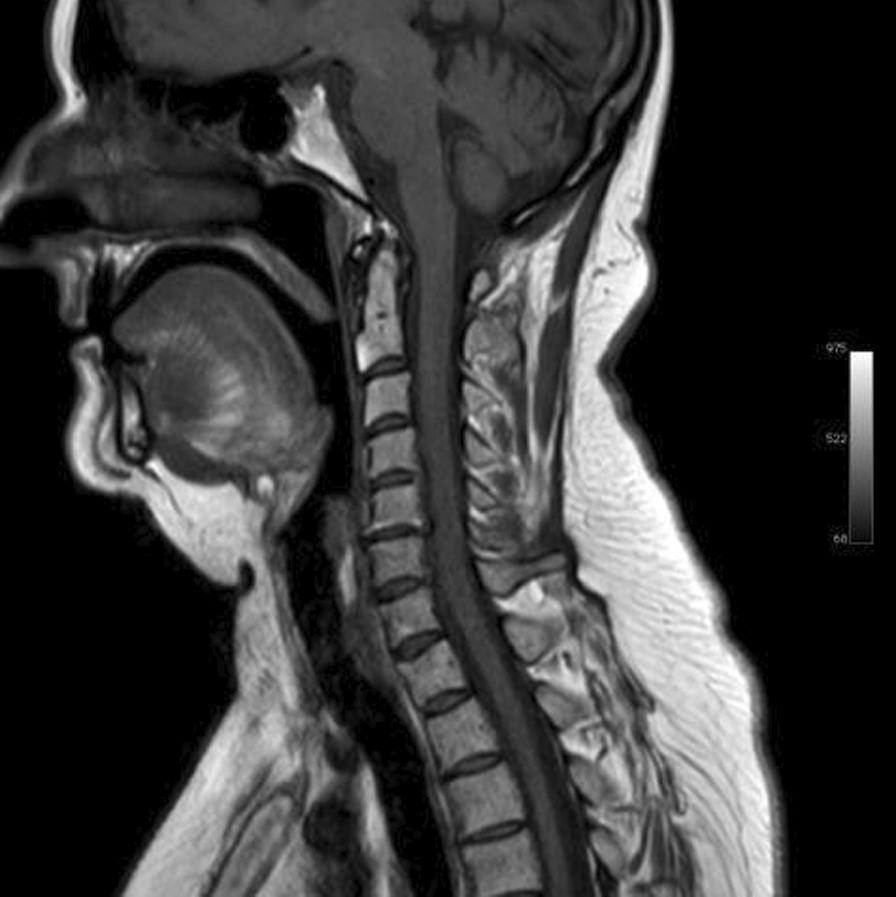
Sagittal T1 demonstrates oedema in the cervico-medullary junction with post contrast enhancement within this region

**Fig. 8:**
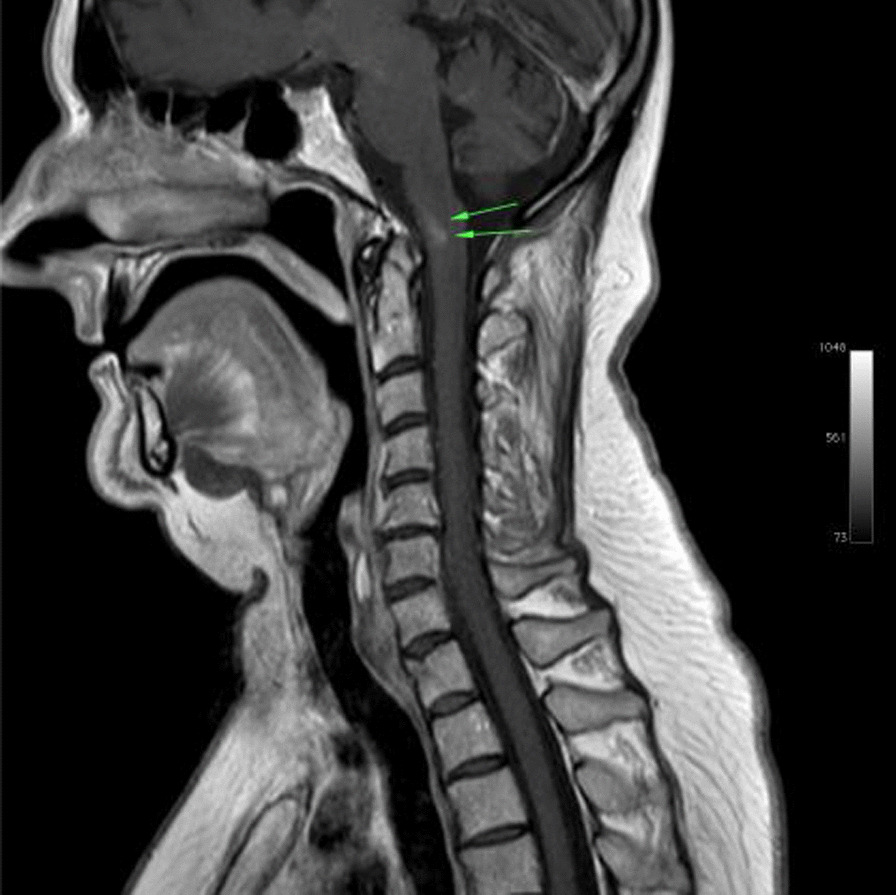
Sagittal T1 after IV Gadolinium demonstrates oedema, as indicated by the arrows, in the cervico-medullary junction with post contrast enhancement within this region

**Fig. 9:**
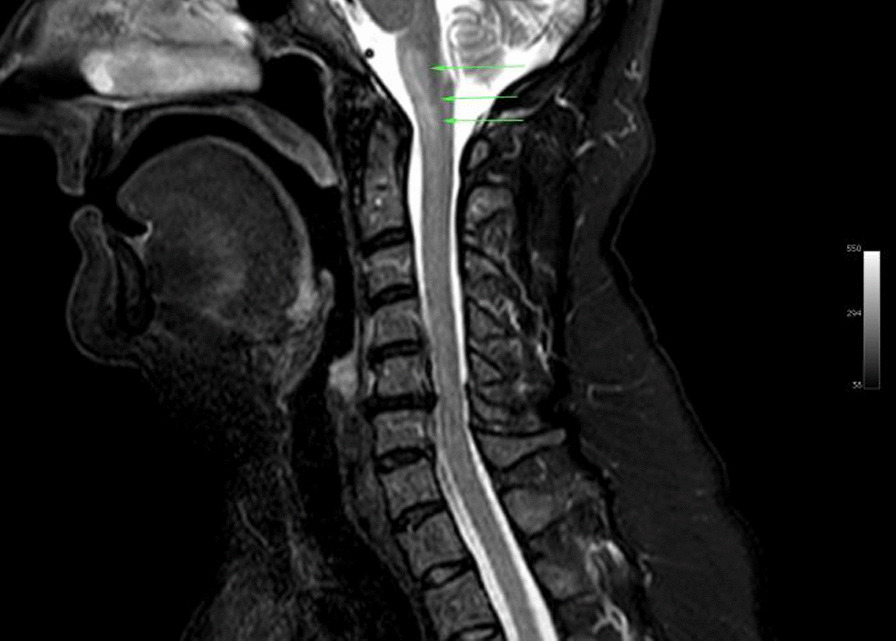
Sagittal STIR demonstrates oedema, as highlighted by the arrows, in the cervico-medullary junction with post contrast enhancement within this region

**Fig. 10:**
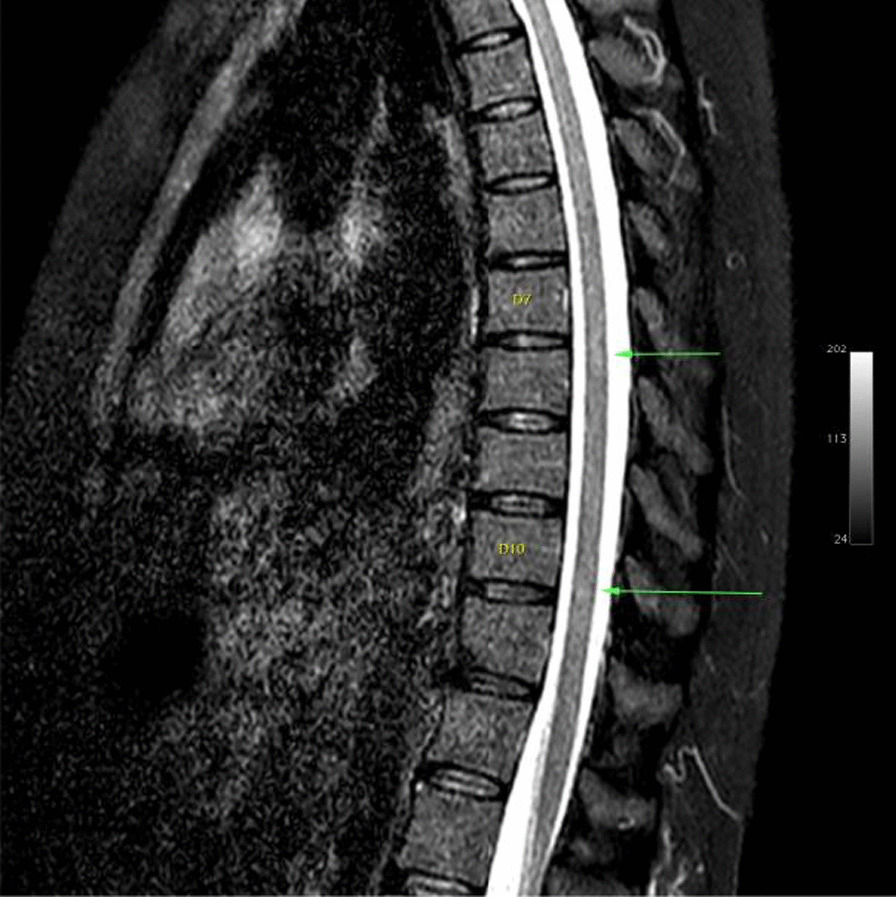
Sagittal STIR of the dorso-lumbar spine demonstrates subtle abnormal increased signal at the D7/8 (upper arrow) and D10/11 levels (lower arrow)

Ms. M spent the next 2 months at home but on the 2 May 2021 she developed significant weakness of her limbs and a generalised seizure was witnessed by her family. She was admitted to our facility and was given a loading dose of intravenous Sodium Valproate and placed on seizure surveillance. Mrs. L. M was noted to have marked weakness of all her limbs, urinary retention and faecal incontinence. An MRI brain and whole spine with gadolinium was ordered which showed prominent bilateral and symmetric hyper-intense T2 AND FLAIR lesions (Figs. 1, 2) in the corticospinal tracts extending to the medullary pyramids, which demonstrated T2 shine-through characteristics on the diffusion weighted sequences (high b1000 signal without commensurate signal loss on the apparent diffusion coefficient map) (Figs. 3, 4). These symmetric findings have, to our knowledge, not yet been previously reported in literature. There were further areas of periventricular and subcortical asymmetric white matter demyelination (Figs. 5, 6) which did not demonstrate any diffusion abnormality. There were no susceptibility changes to suggest any calcification or hemorrhage and the arterial system and dural venous sinuses were normal.None of these supratentorial lesions demonstrated any post contrast enhancement. There was however, a further lesion within the spinal cord at the cervico-medullary junction (Figs. 7, 8, 9) which did demonstrate post contrast enhancement. There were further non-enhancing lesions of the spinal cord noted at D7/8 and D10/11 (Fig. 10). There were background incidental degenerative changes of the spine. A diagnosis of acute post viral disseminated encephalomyelitis (ADEM) was made with further differential for the symmetric intracranial changes of amyotrophic lateral sclerosis being considered.

A lumbar puncture was performed on the 03 May 2021 which showed an elevated protein (502 mg/L), a marginally increased CSF IgG of 35.2 with no polymorphs or red blood cells.

An anti NMDA receptor antibody was repeated and she was commenced on Solumedrol 500 mg IVI daily for 3 days. The response to steroids was initially promising but thereafter she continued to weaken.

The anti NMDA receptor antibody titre was again positive with a titre of 1:640. It was decided, in light of the positive antibody result and clinical deterioration to offer Ms. M plasmapheresis which was commenced on the 07 May 2021.

Ms. M received 5 sessions of plasma exchange and thereafter a repeat dose of IVIG (2 g/kg over 5 days) was administered. Rigorous physiotherapy was undertaken and she began to improve with regards to her strength and mobility. Ms. M was seen by a psychiatrist who switched the escitalopram and trazodone to vortioxetine. She was discharged home on oral prednisone 40 mg daily and advised to repeat the dose of IVIG in 4 weeks. No adverse events were reported prior to discharge.

Ms. M returned in 4 weeks for her repeat dose of IVIG and had improved significantly from her admission on the 2 May 2021. A discussion on the mode of immunosuppressive/modulator therapy was undertaken and the use of rituximab is being considered as long term therapy.

It is thought that due to the fact she has had a severe relapse twice before Ms. M will require chronic therapy. She continues to receive her immunoglobulin therapy every 4 weeks and has shown steady improvement. Her anti NMDA receptor antibody titres will be monitored to assess, along with her clinical improvement, the response to therapy.

Ms. M, after careful consideration, was advised to vaccinate against COVID-19. She received her Pfizer vaccine 6 weeks apart and this was timed to be given about a week prior to receiving her Immunoglobulin dose to allow her to be monitored for any worsening of her neurological condition. It was also considered beneficial to having the immunoglobulin therapy after to mitigate any potential autoimmune flare.

## Discussion

Neurological complications of COVID-19 have been steadily reported since the pandemic began in late 2019. A UK-wide surveillance study performed in the first half of 2020 [1] revealed a multitude of neurological complications in a 153 cases ranging from cerebrovascular events to altered mental status. Those with altered mental status were classified as having, encephalitis, unspecified encephalopathy or a psychiatric condition.

In fact, more than 80% of patients admitted with with COVID-19 to a hospital in Chicago have displayed some form of neurological manifestation [2].

A case series comprising of 3 patients in a hospital in Minnesota, USA commented on all 3 patients having MRI changes and a CSF showing elevated protein indicating ADEM post COVID [3].

Dalmau *et al.*, in 2007, were the first to describe neurological and psychiatric symptoms in women with ovarian teratomas [4]. They discovered an immunoreactivity against the N-methyl D-aspartate receptor (NMDA) 1 subunit of the NMDA receptor in these patients which resulted in the neurological symptoms. Anti NMDA receptor (anti-NMDAR) encephalitis is considered amongst the most common types of autoimmune encephalitis and since the link was discovered there have been multiple new cases described since 2007.

Specifically with regards to COVID-19, a literature review revealed two other case studies in which patients with COVID-19 developed an Anti NMDA receptor encephalitis. Panariello *et al.* presented a young male with symptoms of COVID-19 as well as disorganised behaviour and thought [5]. He progressed to become encephalopathic and was noted to have antibodies to the NMDA receptor. His condition stabilised and began to improve after treatment with IV dexamethasone and intravenous immunoglobulin.

Monti *et al.* presented a 50 year old male patient with fever, psychiatric symptoms and progression to focal seizures with refractory status epilepticus [6]. Subsequent tests revealed anti-NMDAR antibodies and he improved with plasma exchange and treatment with intravenous immunoglobulin therapy.

The course of an autoimmune encephalitis may lead to a misdiagnosis in certain cases. The initial phase is typical of a viral flu-like illness which may progress to hallucinations and agitated behaviour. This may lead to the misdiagnosis of a primary psychiatric condition. Thereafter the patient may progress to developing seizures, catatonia, inattention and dyskinesia [7].

The link between COVID-19 and the development of anti NMDA receptor encephalitis has not been well described but it is considered to be on the basis of the a hyper-inflammatory state triggered by the COVID-19 virus which causes the release of cytokines. These cytokines trigger or activate the glial cells which may result in the demyelination noted in ADEM [8, 9].

There are currently no guidelines that exist on the management of anti NMDA receptor encephalitis in COVID patients. The duration and course of the disease has not been well established and the frequency of flares has not been established. Treatment of NMDAR encephalitis consists of immunotherapy which may involve the use of corticosteroids, intravenous immunoglobulin therapy or plasma exchange [10]. Our patient was treated with all three modalities in order to obtain control of the progression of symptoms and, in light of the fact she has already had 2 previous relapses, she will need to be closely monitored over the next few months as she continues with therapy.

A previous case study in an adolescent male with anti-NMDA receptor encephalitis (not related to COVID) gives credence to the use of immunoglobulin therapy followed by the use of rituximab [11]. In this case the patient showed signs of improvement 5–7 days after completing the first does of intravenous immunoglobulin therapy. A repeat dose was given a month later when his hitherto ongoing improvement had stalled. He was treated with rituximab 14 weeks after admission in 2 divided doses of a 1000 mg each 2 weeks apart with a view to prevent relapse. He only returned to baseline function 3 months after discharge.

In Dalmau’s initial case series three quarter’s of the patients with NMDA receptor antibodies recover fully or have mild outcomes while a quarter will demise or have severe deficits [12].

In a series of patients with anti- NMDA receptor encephalitis presented by Finke *et al.* [13] those who were treated early with immunotherapy performed significantly better than those who had delayed initiation of appropriate therapy. Early treatment was especially important in limiting permanent cognitive deficits.

The MRI changes are an important factor in establishing the diagnosis. The presence of multifocal, bilateral asymmetric areas of oedema or demyelination (increased T2 and FLAIR weighted signal lesions), of the brain and spinal cord, which may or may not enhance and may or may not demonstrate restricted diffusion in a patient who has a recent history of a viral illness, is key to the diagnosis of ADEM [14]. The finding, in our case, of bilateral corticospinal tract involvement was not typical of classic ADEM. The lesions may be multifocal and punctate or large and confluent (tumefactive lesions). The grey matter of the basal ganglia is usually involved, although less commonly, as is the spinal cord [15]. MRI is currently the most sensitive and specific investigation for ADEM [14], particularly when compared with CT, which in some cases may be normal in the presence of significant disease. The main differential diagnosis to consider would be that of Multiple Sclerosis which more commonly involves the calloso-septal interface and usually does not involve the brainstem [16].

## Conclusion

The arrival of the COVID-19 Pandemic has resulted in a plethora of new associated disease presentations. Acute demyelinating encephalomyelitis is a debilitating complication of the viral disease. It is important to recognise the symptoms of ADEM and perform the appropriate investigations in a timeous manner in order to confirm a diagnosis and initiate early treatment to limit permanent neurological deficits. In particular, with regards to MRI findings, COVID-19 ADEM may present with unusual imaging findings as evidenced by the bilateral symmetric involvement of the corticospinal tract as noted in our case.

## Data Availability

Patient records are kept onsite in the treating doctors patient file.

## Abbreviations

ADEM: Acute demyelinating encephalomyelitis
NMDA: N-methyl-d-aspartate
MRI: Magnetic resonance imaging
IVIG: Intravenous immunoglobulin
IV: Intravenous
